# Wissenschaftskommunikation zwischen Gesellschafts‑, Wissenschafts- und Medienwandel

**DOI:** 10.1007/s11616-020-00623-2

**Published:** 2020-11-23

**Authors:** Birte Fähnrich, Mike S. Schäfer

**Affiliations:** 1 Berlin-Brandenburg Academy of Sciences and Humanities, Jägerstraße 22/23, 10117 Berlin, Deutschland; 2 IKMZ – Institut für Kommuniationswissenschaft und Medienforschung, Universität Zürich, Andreasstrasse 15, 8050 Zürich, Schweiz

## Wissenschaftskommunikation als dynamisches Handlungsfeld

Kaum ein Thema hat die Wissenschaft in den vergangenen Jahren disziplinübergreifend und international so beschäftigt wie die Wissenschaftskommunikation. Das galt schon vor der gegenwärtigen COVID-19-Pandemie und gilt heute umso mehr: Einzelne Wissenschaftlerinnen und Wissenschaftler, aber auch Hochschulen und Wissenschaftsorganisationen sind medial zunehmend präsent. Wissenschaftspolitik, Förderorganisationen und Fachverbände fordern und fördern die öffentliche Kommunikation über wissenschaftliche Themen in Journalismus und Social Media, den Auftritt wissenschaftlicher Akteure in öffentlichen Debatten sowie mehr kommunikatives Engagement von Wissenschaftlerinnen und Wissenschaftlern (vgl. überblicksweise Bonfadelli et al. 2017; Fähnrich et al. 2019; Schäfer et al. 2015). Dem wissenschaftspolitischen Diskurs liegen dabei unterschiedliche normative Vorstellungen zugrunde. So fordert das 2019 erschienene Grundsatzpapier des Bundesministeriums für Bildung und Forschung zur Wissenschaftskommunikation einen „Kulturwandel hin zu einer kommunizierenden Wissenschaft“ (BMBF 2019, S. 2) und betont damit einen spezifischen Bereich von Wissenschaftskommunikation: „organized actions aiming to communicate scientific knowledge, methodology, processes or practices in settings where non-scientists are a recognised part of the audience“ (Davies und Horst 2016, S. 4). Kommuniziert werden solle v. a. *aus* der Wissenschaft heraus, durch individuelle Forschende, Medienabteilungen oder Leitungen von Hochschulen und Forschungseinrichtungen, und im Rahmen vielfältiger Online- und Offline-Formate. Im Mittelpunkt steht dabei der Dialog von Wissenschaft und Gesellschaft. Public Understanding of Science, Public Engagement, Citizen Science oder auch Open Science sind Schlagworte, die seit einigen Jahren als „Goldstandard“ für diese Interaktionen gelten (vgl. Fähnrich 2017; Felt und Fochler 2008; Bucchi und Trench 2016).

Mit einem holistischen, stärker analytischen Verständnis lässt sich Wissenschaftskommunikation aber breiter fassen. Denn für die öffentliche Wahrnehmung von Wissenschaft kann grundsätzlich jede wissenschaftsbezogene Kommunikation eine wichtige Rolle spielen. Dabei sind nicht nur die Eigenkommunikation der Wissenschaft oder die journalistische Berichterstattung über Wissenschaft relevant. Wissenschaftliche Themen sind zunehmend auch Gegenstand der Kommunikation zahlreicher anderer gesellschaftlicher Akteure wie politischer Parteien, Unternehmen, NGOs oder Think Tanks. Diese kommunizieren auf Online-Kanälen und Plattformen aus verschiedenen Gründen *über* wissenschaftliche Themen, wobei sie diese nicht selten popularisieren, instrumentalisieren, entertainisieren, emotionalisieren oder dekontextualisieren (vgl. z. B. Mede und Schäfer 2020; Fähnrich 2018; Allgaier 2017; Kahan et al. 2017).

Ein umfassendes Verständnis von Wissenschaftskommunikation schließt zudem Kommunikation *in* der Wissenschaft, also zwischen wissenschaftlichen Peers, ein. Das gilt umso mehr, als durch die Digitalisierung Grenzen zwischen inner- und außerwissenschaftlicher Kommunikation hybrider und poröser werden, wissenschaftliche Debatten zum Beispiel via Twitter von einem breiten Publikum mitverfolgt werden können oder wissenschaftliche Kommunikate durch Open-Science-Standards auch für ein breites (Laien‑)Publikum zugänglich sind (vgl. z. B. Jünger und Fähnrich 2019; Neuberger 2014). Dem vorliegenden Themenheft liegt ein solch breites Verständnis von Wissenschaftskommunikation zugrunde. Gemeint sind also „alle Formen von auf wissenschaftliches Wissen oder wissenschaftliche Arbeit fokussierter Kommunikation, sowohl innerhalb als auch außerhalb der institutionalisierten Wissenschaft inklusive ihrer Produktion, Inhalte, Nutzung und Wirkung“ (Schäfer et al. 2015, S. 13). Wissenschaftskommunikation – in dieser Weise verstanden – hat sich in den vergangenen Jahrzehnten deutlich gewandelt. Dies ist eine Begleiterscheinung umfassender und eng miteinander verknüpfter Wandlungsprozesse von Gesellschaft, Medien und Wissenschaft selbst (vgl. Weingart 2005). Die Kommunikation in, von und über Wissenschaft ist von diesen Wandlungsprozessen grundlegend betroffen und wirkt zugleich auf diese zurück.

So wird mit der Entwicklung der „Wissensgesellschaft“ (Stehr 1994) wissenschaftliches Wissen vielfach als bestmögliches Wissen angesehen: „The ideological value of science lies in its great prestige as an arbiter of what is true and what is false, of what is real and what is not real.“ (Shepherd 1981, S. 131) Wissenschaftskommunikation – so scheint es – trägt damit dynamisch zur Entwicklung der modernen Gesellschaft bei. Ursache und zugleich Ergebnis dieses Prozesses ist, dass Wissenschaft zunehmend öffentlich sichtbar wird – ein Trend, den die Digitalisierung der öffentlichen Kommunikation wie oben bereits beschrieben massiv befördert. Andererseits lassen sich entgegengesetzte Effekte beobachten. So verschieben sich im Kontext des Medien- und Öffentlichkeitswandels gesellschaftliche Wissensordnungen (vgl. Neuberger et al. 2019). Bereits unter dem Schlagwort der „Risikogesellschaft“ (Beck 1986) wurde Wissenschaft zunehmend mit Unsicherheit, Kontingenz und Risiken in Verbindung gebracht und konnte „weniger gut den Status als dominanter relevanter Wissensproduzent in der Gesellschaft behaupten“ (Görke und Rhomberg 2017, S. 44). Im „postfaktischen Zeitalter“ (Berling und Bueger 2017) hat sich daraus eine Krisendiagnose entwickelt, die für traditionelle epistemische Autoritäten wie Journalismus und Wissenschaft gleichermaßen gilt, wenn auch in unterschiedlichem Maße.

Die Digitalisierung öffentlicher Kommunikation wird als eine der zentralen Ursachen für die Erosion einer gemeinsamen gesellschaftlichen Wissensbasis gesehen. Auch wenn das gesellschaftliche Vertrauen in Wissenschaft insgesamt hoch ist (vgl. z. B. Dommett und Pearce 2019), lässt sich an der Formierung wissenschaftsskeptischer (Gegen‑) Öffentlichkeiten, etwa im Kontext von Klimawandel, Gentechnologie und Impfdiskurs, die Verschiebung öffentlicher Rationalitäten nachzeichnen (vgl. Neuberger et al. 2019). Dies trägt wiederum zu verstärkten Forderungen nach *mehr* Wissenschaftskommunikation bei (vgl. Ruhrmann und Guenther 2017). Die Entwicklungen in Gesellschaft und Medien und damit einhergehende Intensivierungen und Veränderungen von Wissenschaftskommunikation verschieben aber auch die Anreizstrukturen und Relevanzordnungen innerhalb der Wissenschaft. Die Öffentlichkeitsorientierung der Wissenschaft hat sich in den vergangenen Jahren verstärkt (vgl. Peters 2019; Ke et al. 2017). Sie wird teilweise problematisiert, „weil mit dem Gang in die Medien Kontrollverlust der innerwissenschaftlichen Qualitätssicherung impliziert ist“ (Pansegrau et al. 2011, S. 4) – eine Entwicklung, die mit den aktuellen Diskussionen über die Verwendung von Preprints zu COVID-19-bezogenen Forschungsergebnissen auf die Spitze getrieben wird. Dennoch machen etwa die Diskussionen um alternative Metriken (vgl. Priem und Hemminger 2010) zur Messung wissenschaftlichen Impacts auch die innerwissenschaftlich steigende Relevanz des sogenannten Public Engagement deutlich; so wird die aktive öffentliche Kommunikation von Wissenschaftlerinnen und Wissenschaftlern zunehmend als „a normative feature of the job“ angesehen (Bauer und Jensen 2011) und scheinen die gesellschaftliche Wahrnehmung und die zugeschriebene Reputation mehr und mehr interdependent (vgl. Hegglin und Schäfer 2015).

## Wissenschaftskommunikation und Kommunikationswissenschaft

Die hier skizzierten Entwicklungen der Wissenschaftskommunikation im Kontext eines umfassenderen Gesellschafts‑, Medien- und Wissenschaftswandels verdeutlichen die Relevanz einer systematischen und kritischen Beobachtung und Reflexion durch die Forschung zur Wissenschaftskommunikation. Dabei handelt es sich um ein dynamisches und wachsendes Forschungsfeld, das sich seit den 1980er-Jahren international und im deutschsprachigen Raum verstärkt seit der Jahrtausendwende entwickelt (vgl. Bauer 2017; Peters et al. 2020). Hier befassen sich eine Reihe von sozial- und geisteswissenschaftlichen Disziplinen mit der öffentlichen Kommunikation in, von und über Wissenschaft (vgl. Rauchfleisch und Schäfer 2018). Die Kommunikationswissenschaft leistet im deutschsprachigen Raum wie international wichtige Beiträge für dieses Forschungsfeld (vgl. Kessler et al. 2019). Sie ist durch ihren zentralen Forschungsgegenstand – die öffentliche und medial vermittelte Kommunikation (vgl. DGPuK 2008) –, durch ihre über Jahrzehnte entwickelten theoretischen und begrifflichen Ansätze und durch ihre methodischen Zugänge prädestiniert dafür, zentrale Fragen zu bearbeiten, die sich im Zusammenhang mit Wissenschaftskommunikation stellen. Kommunikationswissenschaftliche Forschung hat somit in den vergangenen Jahren zu einer Reihe von theoretisch-konzeptionellen Modellen einerseits und instruktiven empirischen Befunden andererseits geführt (im Überblick vgl. Schäfer et al. 2015, 2020; Bonfadelli et al. 2017; Fähnrich et al. 2019), die helfen, die Entwicklungen der Wissenschaftskommunikation zu beobachten, zu bewerten und punktuell zu prognostizieren.

Umgekehrt kann die Forschung zu Wissenschaftskommunikation auch die Entwicklung der Kommunikationswissenschaft selbst beleuchten. Denn die öffentliche Sichtbarkeit des Fachs wird insgesamt kritisch bewertet, wenngleich bislang nur wenige Studien zur Wissenschaftskommunikation der Kommunikationswissenschaft vorliegen. So zeigen Brantner und Huber (2013), dass wenige Kommunikationswissenschaftler und Kommunikationswissenschaftlerinnen im deutschsprachigen Raum in Printmedien zu Wort kommen. Nielsen (2018) behauptet für die Politische Kommunikationsforschung gar „no one cares what we know“ und begründet die geringe Sichtbarkeit des Fachs auch mit der Selbstbezüglichkeit seiner Wissenschaftlerinnen und Wissenschaftler – eine These, die sich empirisch zumindest teilweise widerlegen lässt (vgl. Jünger und Fähnrich 2019). Entsprechend lohnt zukünftige systematische Forschung zur Wissenschaftskommunikation der Kommunikationswissenschaft, weil sie zeigen könnte, mit welchen Themen das Fach in die Gesellschaft tritt, als wie wichtig es von außen wahrgenommen wird, wie sich diese Faktoren im Zeitverlauf verändern, wie das Fach im Vergleich zu anderen Disziplinen steht und gegebenenfalls auch, welche Implikationen dies für die Interaktion von Kommunikationswissenschaft, Politik und Gesellschaft selbst hat.

Die *Publizistik* eignet sich wie kaum ein anderer Publikationsort im deutschsprachigen Raum für eine solche Bestandsaufnahme der kommunikationswissenschaftlichen Forschung zur Wissenschaftskommunikation und die gleichzeitige Selbstreflexion der kommunikationswissenschaftlichen Wissenschaftskommunikation. Als älteste deutschsprachige Fachzeitschrift feiert die *Publizistik* in diesem Jahr einen runden Geburtstag: Sie ist seit 65 Jahren ein Forum für Beiträge aus Kommunikationswissenschaft und Medienforschung und Sinnbild für den über Dekaden akkumulierten theoretischen und methodischen Erfahrungsschatz des Faches. Zudem war und ist sie auch immer wieder der Ort für disziplinäre Selbstverständnisdebatten, etwa mit Blick auf den Gegenstand des Faches (vgl. Brosius 2016; Hepp 2016; Jarren 2016), seine künftige Entwicklung (vgl. Strippel et al. 2018) oder seine paradigmatische Ausrichtung (vgl. Schäfer und Wessler 2020).

Vor diesem Hintergrund ist es Ziel des vorliegenden Themenhefts, einerseits Einblicke in das Forschungsfeld Wissenschaftskommunikation zu geben. Andererseits bietet das Themenheft aber auch eine Plattform für disziplinäre Rückschau und Selbstreflexion. Der aktuelle Informationsbedarf rund um die Corona-Pandemie, aber auch die neueren wissenschaftspolitischen Entwicklungen verdeutlichen die Relevanz des Themenfelds und die Notwendigkeit seiner kritischen Beobachtung aus wissenschaftlicher Perspektive nochmals eindrücklich. Die Kommunikationswissenschaft hat hier die Chance, nachhaltig ein hochrelevantes Thema zu besetzen und damit auch ihre strategische Rolle im Konzert der Disziplinen zu verdeutlichen.

## Beiträge des Themenhefts

Das Themenheft gibt einen Einblick in aktuelle kommunikationswissenschaftliche Forschung zu Wissenschaftskommunikation und stellt Studien zur Wissenschaftskommunikation der Kommunikationswissenschaft selbst vor.

*Erik Koenen* und *Juliane Pfeiffer* behandeln in ihrem Beitrag „*Dovifats Dias*“ zunächst „*Historische Perspektiven der Wissenschaftskommunikation der Kommunikationswissenschaft*“. In ihrer historischen Analyse setzen sie sich kritisch mit den öffentlichen Transfer- und Vermittlungsleistungen unserer Disziplin auseinander und analysieren, welche Rolle diese in der Fachgeschichte gespielt haben. Sie zeigen, dass Wissenschaftskommunikation in der Kommunikationswissenschaft historisch wenig Aufmerksamkeit erhalten hat. Jedoch gab es im Laufe der Fachentwicklung prägende Akteure, deren Analyse für die Selbstreflexion des Fachs lohnenswert erscheint. Einen dieser Akteure, den Gründungsvater der Zeitungs- und Publizistikwissenschaft Emil Dovifat, beschreiben Koenen und Pfeiffer in ihrer Retrospektive als engagierten und erfolgreichen Wissenschaftskommunikator.

Eine aktuelle Bestandsaufnahme der Kommunikation über die Kommunikationswissenschaft liefern *Silke Fürst, Daniel Vogler, Isabel Sörensen, Mike S. Schäfer* und *Mark Eisenegger*. In ihrem Beitrag „*Wirklich irrelevant? Sichtbarkeit und thematische Einordnung der Medien- und Kommunikationswissenschaft in Schweizer Medien*“ untersuchen sie die mediale Wahrnehmung des Faches in der Schweiz. Sie legen eine automatisierte Inhaltsanalyse zur Mediensichtbarkeit und thematischen Einordnung des Faches auf Basis einer Vollerhebung der Berichterstattung sieben überregionaler Zeitungen von 1999 und 2018 vor. Ihre Befunde zeigen, dass die öffentliche Sichtbarkeit der Kommunikations- und Medienwissenschaft hinter anderen sozialwissenschaftlichen Fächern zurücksteht und dass dieser Rückstand im Zeitverlauf noch zunimmt. Interessant ist zudem, dass über die Kommunikationswissenschaft zwar im Kontext verschiedener Themen, aber selten unter Bezug auf das Thema Digitalisierung berichtet wird. Mit ihrer Defizitdiagnose sprechen sich die Autorinnen und Autoren für Maßnahmen zur Stärkung der gesellschaftlichen Sichtbarkeit und Legitimation des Fachs aus.

Zwei weitere Aufsätze wenden sich nicht der Kommunikationswissenschaft, sondern der Schnittstelle zwischen Gesellschafts‑, Wissenschafts- und Medienwandel allgemein zu. Dazu gehört der Beitrag von *Stefanie Walter, Janne Görlach* und *Michael Brüggemann* zum Thema Klimajournalismus mit dem Titel „*Climate Feedback: Wissenschaft kommentiert Journalismus und entwickelt Mehrsystemkompetenz*“. Die Studie analysiert das Verhältnis von Wissenschaft und Medien mit dem Konzept der Medialisierung der Wissenschaft und untersucht, inwieweit sich die Wissenschaft an Medienlogiken anpasst. Vor diesem Hintergrund untersuchen die Autorinnen und Autoren, welche Kriterien Klimawissenschaftlerinnen und -wissenschaftler bei der Beurteilung journalistischer Artikel heranziehen und wie sie die journalistische Vermittlungsleistung und den wissenschaftlichen Informationsgehalt von Beiträgen gegeneinander abwägen und bewerten. Die Studie umfasst eine qualitative Inhaltsanalyse des Blogs „Climate Feedback“ und bezieht sich auf 82 Blogeinträge und 185 Kommentare im Zeitraum von 2015 bis 2017. Die Ergebnisse legen nahe, dass Forschende bei der Bewertung die Kommunikationsnormen von Wissenschaft und Journalismus kombinieren und damit an der Schnittstelle zwischen Journalismus und Wissenschaft Mehrsystemkompetenz erwerben und anwenden können.

Schließlich nehmen *Anne Hennig* und *Sarah Kohler* die Online-Kommunikation von Wissenschaftlerinnen und Wissenschaftlern in den Blick und analysieren „*Einflussfaktoren auf die Social-Media-Nutzung in der Wissenschaftskommunikation*“. Dabei geht es um die Frage, welche Faktoren die Nutzung sozialer Medien durch Forschende beeinflussen und damit auch, welche Akteure auf sozialen Medien besonders sichtbar sind und dort Wissenschaft repräsentieren. Die Studie, die teils als Replikation vorangehender Untersuchungen angelegt ist, untersucht die Häufigkeit der Nutzung von Facebook, Twitter, Instagram, YouTube, Snapchat sowie von Blogs und Podcasts von 1100 Wissenschaftlerinnen und Wissenschaftlern. Die Ergebnisse einer binär-logistischen Regressionsanalyse bestätigen die Befunde bisheriger Studien dabei nur punktuell. Gerade mit Blick auf die Relevanz der Online-Kommunikation von Wissenschaftlerinnen und Wissenschaftlern für die öffentliche Wahrnehmung verweisen die Autorinnen daher auf die Notwendigkeit von Anschlussforschung, um ein differenzierteres Bild der Motivationen zur (Nicht‑)Nutzung von Social Media zeichnen zu können.
